# Misdiagnosis of Calcaneal Chondrosarcoma as Plantar Fasciitis: A Case Report

**DOI:** 10.7759/cureus.93686

**Published:** 2025-10-02

**Authors:** Kia Teng Lim, Zakareya Gamie, Natalie Green, Rajesh Botchu, Petra Balogh, Vineet Kurisunkal, Scott Evans

**Affiliations:** 1 Orthopaedic Surgery, National University Hospital, Singapore, SGP; 2 Orthopaedic Oncology, Newcastle upon Tyne Hospitals NHS Foundation Trust, Newcastle, GBR; 3 Orthopaedic Oncology, Royal Orthopaedic Hospital, Birmingham, GBR; 4 Radiology, Royal Orthopaedic Hospital, Birmingham, GBR; 5 Histopathology, Queen Elizabeth Hospital, Birmingham, GBR

**Keywords:** bone, calcaneus, chondrosarcoma, fasciitis, plantar, sarcoma, surgery

## Abstract

Chondrosarcoma is the most common malignant bone tumour amongst the elderly. However, they often present as diagnostic dilemmas leading to diagnostic delays, leading to poor prognosis in elderly patients. Chondrosarcoma of the foot is also exceedingly rare, comprising <6% of bone tumors, where its clinical presentation can mimic common soft tissue conditions such as plantar fasciitis. We report the case of a 79-year-old female who presented with non-mechanical right foot pain involving the heel, initially misdiagnosed as plantar fasciitis for two years. Her foot pain remained recalcitrant to conservative management. Advanced imaging performed revealed a large lytic lesion in the calcaneus, initially thought to represent an aneurysmal bone cyst. Confirmatory pre-operative biopsy revealed a calcaneum chondrosarcoma. Below-knee amputation (BKA) was performed, and the outcome was good, with no reported local recurrence or metastases. Bone malignancy should be considered as a key differential in all cases of non-mechanical pain. As it may be challenging to clinically differentiate between recalcitrant plantar fasciitis and other malignant conditions of the foot, imaging should be mandatory for all patients presenting with non-mechanical pain.

## Introduction

Chondrosarcoma is the most common malignant bone tumour in the elderly population, accounting for approximately 30% of all primary bone tumours [1]. Foot chondrosarcoma can be as rare as one in 10,000 cases described in early reports [2]. Patients with chondrosarcomas generally have favourable survival outcomes, with an overall 5-year survival rate of approximately 75% [3]. However, the prognosis of elderly patients with chondrosarcomas remains poor, with elderly patients showing significantly lower 5-year overall survival (OS) compared to younger patients [4]. In the analysis of over 2800 patients from the Surveillance, Epidemiology, and End Results (SEER) database, patients who were ≤ 50 years old were found to have a significantly higher disease-free survival and OS compared to those >50 years old [5].

Chondrosarcomas are also known to present diagnostic dilemmas, which may invariably lead to diagnostic delays, particularly in differentiating malignant low-grade tumours from benign enchondromas, and differentiating between high and intermediate grade tumours [6]. Several nationwide guidelines have been implemented to streamline cancer diagnosis waiting times - most recently in the United Kingdom, the Faster Diagnosis standard was initiated in a few regions in accordance with the clinically-led review of National Health Service (NHS) access standards (CRS) [7], with a target to diagnose cancers within 28 days of referral; According to the Dutch Stichting Oncologische Samenwerking (SONCOS) guidelines implemented in the Netherlands, the acceptable referral clinic doctor-related delay for a Dutch oncological center to diagnose a neoplasm and commence treatment is 42 days [8]. However, issues pertaining to pre-referral diagnostic delays remain largely unexplored.

In a recent retrospective study by Goedhart et al, the mean delay in diagnosis in patients with chondrosarcoma was found to be 688.0 days, which was significantly longer than diagnostic delays in other bone tumours such as osteosarcoma and Ewing sarcoma [9]. Most of the delays occurred in the pre-hospital stage, where there was a mean 197.2 days' delay between patients’ presentation to the general practitioner (GP) and presentation to a primary hospital. One of the hypothesized reasons for the pre-hospital delay is the rarity of the first presentation of bone malignancies in the GP setting, which may result in unfamiliarity, for which there was a call for GPs to have lower thresholds in performing plain radiographs for patients with atraumatic, persistent pain for more than 6 weeks.

With the advent of increased magnetic resonance imaging (MRI) usage, the annual incidence of chondrosarcoma has shown a marked increase of 68% over the past 30 years, possibly revealing issues with underdiagnosis in the past [10]. The location of the central cartilage tumours in the appendicular skeleton also play a major role in diagnostic delays, with different proportions of benign tumors (enchondromas) to malignant masses (atypical cartilaginous tumours to higher grade chondrosarcomas) across various sites of origin [11].

Malignant tumours of the foot are rare, comprising less than 6% of all bone tumours [12]; in particular, chondrosarcoma of the foot is extremely rare, with only case series and case reports described in literature [13]. The most common symptom at presentation for chondrosarcomas is pain, often described as dull in character and present for months, and rarely a palpable swelling [14].

Plantar fasciitis is one of the most common causes of heel pain, accounting for more than 1 million patient visits per year in the United States of America. It has also been found that up to 44% of patients with plantar fasciitis still have persistent pain 15 years after diagnosis and treatment [15]. Due to the rare occurrence of foot malignancy and common conditions such as plantar fasciitis being known to cause stubborn heel pain, chondrosarcomas are rarely considered as diagnoses for recalcitrant foot pain [16]. Delays in considering additional diagnoses may also be attributed to how diagnostic imaging is often deemed unnecessary for clinical diagnosis of plantar fasciitis [17]. In a retrospective study on the orthopaedic oncology database at Royal Orthopaedic Hospital, Birmingham, chondrosarcomas affecting the foot were also found to be diagnosed later compared to other tumour types, with a median duration of symptoms for 104 weeks [18], which could be attributed to the slow-growing nature of chondrosarcomas.

The current study reports a rare case of a patient with chondrosarcoma of the calcaneus which was misdiagnosed and treated as plantar fasciitis in the primary care setting.

## Case presentation

This patient was a 79-year-old woman who was referred to the Bone Tumour Service in Birmingham. She had been on follow-up with her GP for a 24-month history of right foot pain involving the heel, initially diagnosed as plantar fasciitis but later found to be calcaneal chondrosarcoma. She had a past surgical history of right total hip replacement in 2009, left total hip replacement in 2019, multiple previous corrections for bilateral hallux valgus, and release of Dupuytren’s contracture of the left hand. She had arthritis affecting multiple joints, for which she was on regular pain medications, including duloxetine. She also had a significant history of smoking approximately six cigarettes per day, which she had recently reduced, and had no history of alcohol use. She had no significant family history of medical conditions, including malignancies, and worked as a homemaker.

The patient had been treated by her GP for right foot plantar fasciitis with foot injections for pain relief. However, her right foot pain persisted. She later presented to the emergency department after two years of persistent right foot pain. On examination, there was swelling of the hindfoot with erythema over the heel to midfoot and tenderness over the right ankle joint on palpation. Her range of motion of the ankle joint was preserved, and her neurovascular status was intact.

**Figure 1:**
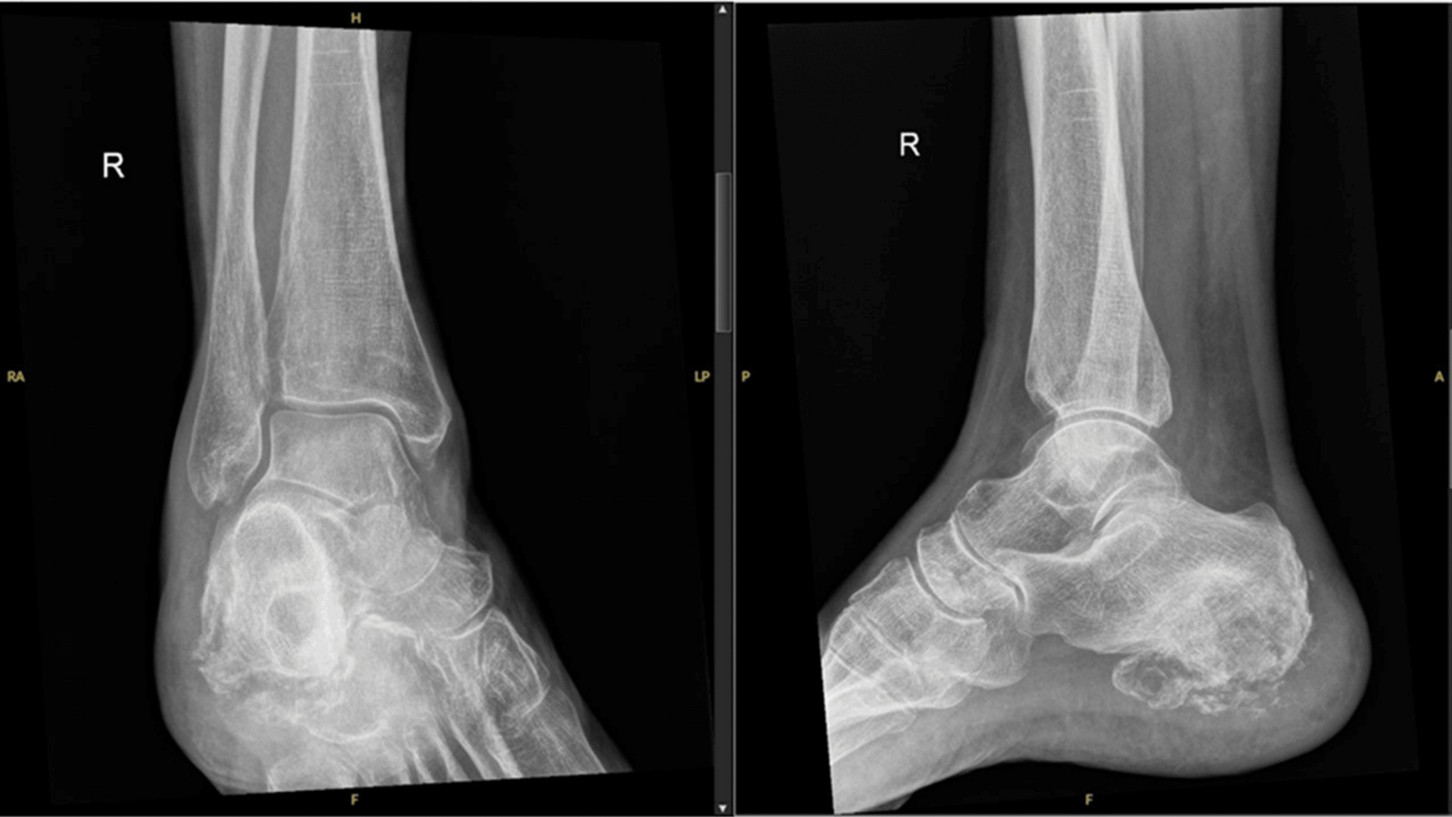
Plain radiographs anterior posterior (AP) and lateral of patient’s right ankle demonstrating a destructive lesion in the calcaneus.

**Figure 2:**
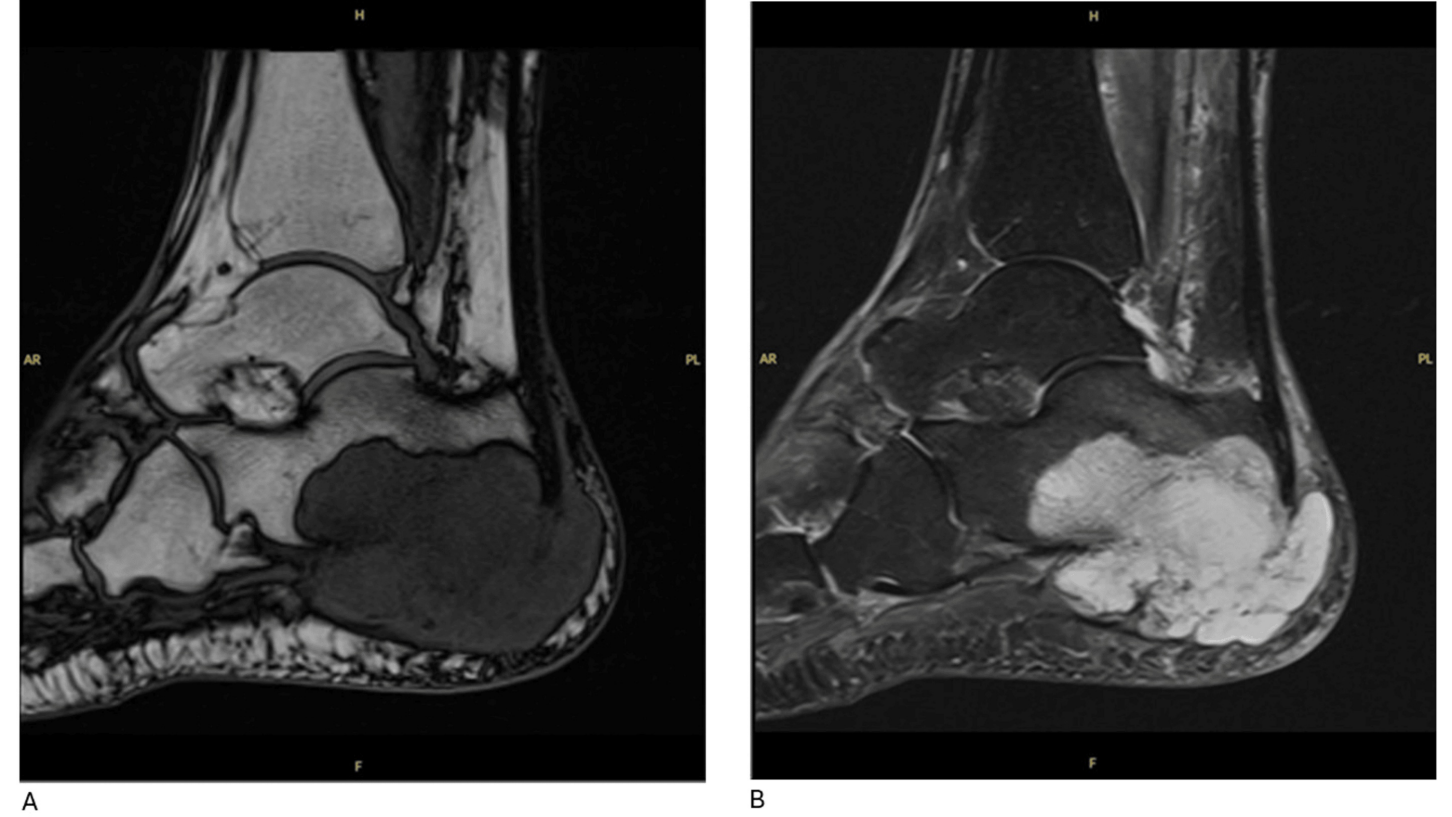
MRI of the patient’s right ankle showing a multiloculated cystic lesion (T1 and STIR sequences). (A) Magnetic Resonance Imaging (MRI) T1 sequence of the patient’s right ankle demonstrating a large multiloculated cystic lesion, (B) Magnetic Resonance Imaging (MRI) Short Tau Inversion Recovery (STIR) T1 sequence of the patient’s right ankle demonstrating the lesion with increased signal intensity.

X-ray (XR) imaging of the right ankle was performed, revealing poor mineralisation of the bones. Fragmentation was also seen, with sclerotic changes on the plantar aspect of the calcaneus posteriorly, suggestive of chronic osseous infection (Figure 1). Contrast MRI of the right foot was also performed, where a large cystic lesion was seen involving the major part of the calcaneum, causing partial cortical destruction. Several bony fragments were also identified alongside the calcaneus contour. Alongside the plantar surface of the calcaneus, adjacent to the plantar fascia insertion and Achilles tendon insertion, a large multiloculated cystic lesion was identified. This lesion demonstrated low intensity on the T1-weighted images and increased signal intensity on the T2-weighted images without enhancement in the contrast sequence (Figures 2A, 2B). The surrounding subcutaneous fat was shown to demonstrate diffuse oedema, and no other isolated abnormal bony signal intensity was identified. The large cystic bony lesion in the MRI was thought to likely represent a calcaneal aneurysmal bone cyst, with biopsy advised due to the destructive bony appearance.

After the imaging scans, this patient presented to the oncology clinic at Royal Orthopaedic Hospital, Birmingham. On examination, she was unable to wear her shoes due to pain. There was also swelling along the calcaneum with erythema corresponding to the site of the lesion. The range of motion of the ankle joint remained preserved. The impression was that of calcaneal chondrosarcoma of the right foot. Treatment options were discussed with the patient; below-knee amputation (BKA) was recommended. Biopsy of the lesion was discussed, with the risk of failure of confirmatory diagnosis of sarcoma on biopsy explained. The patient was keen to proceed with a confirmatory biopsy pre-surgery.

Investigations

Staging scans with a computed tomography (CT) of the chest, abdomen, and pelvis were performed. The CT thorax showed indeterminate pulmonary nodules. CT-guided biopsy of the right foot lesion was also arranged.

**Figure 3:**
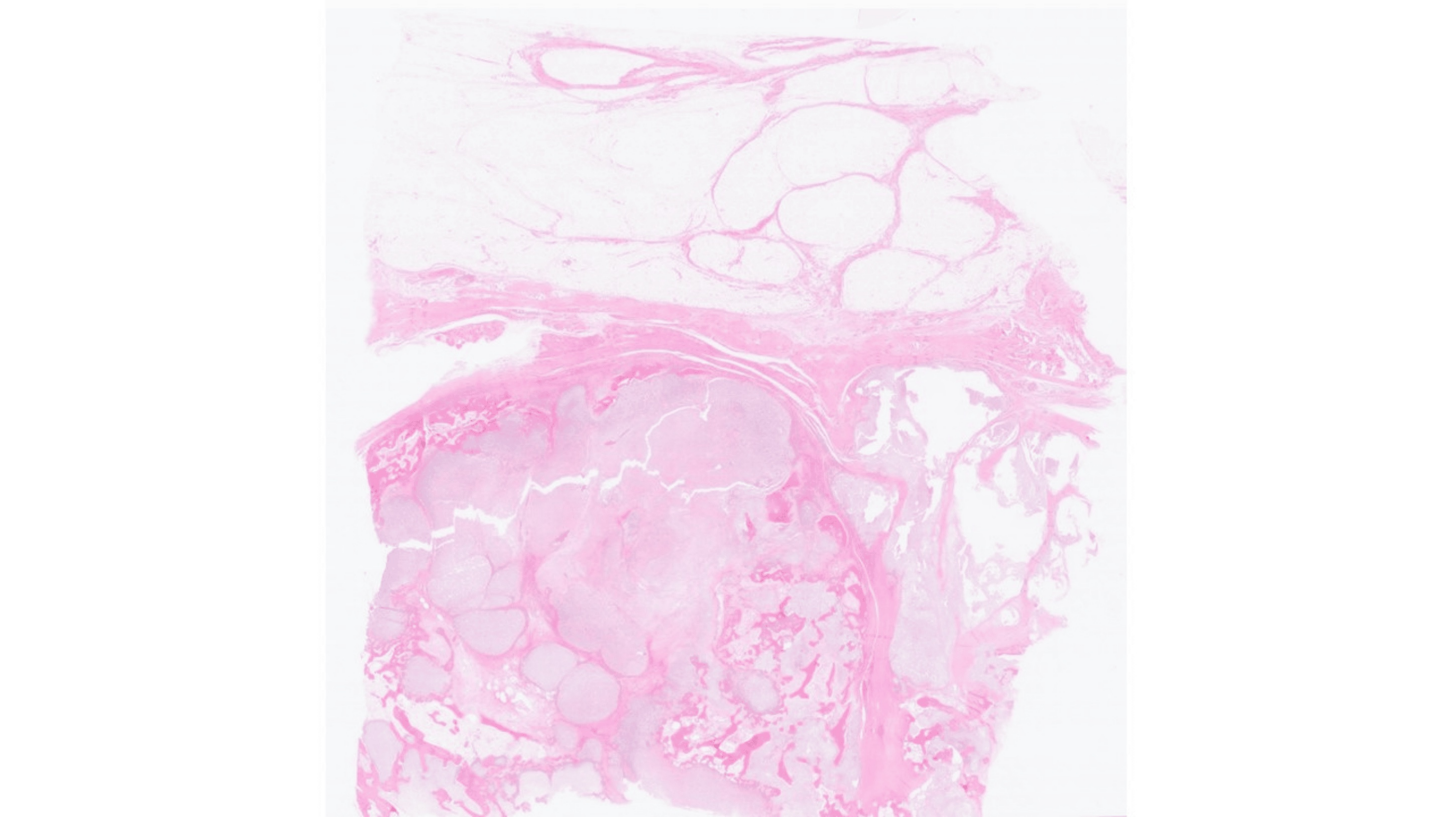
Low power Hematoxylin and Eosin (HE) section shows multinodular cartilaginous tumour infiltrating host bone and showing pushing pattern of growth towards fibroadipose tissue. Magnification, digital slide: 03/43x

**Figure 4:**
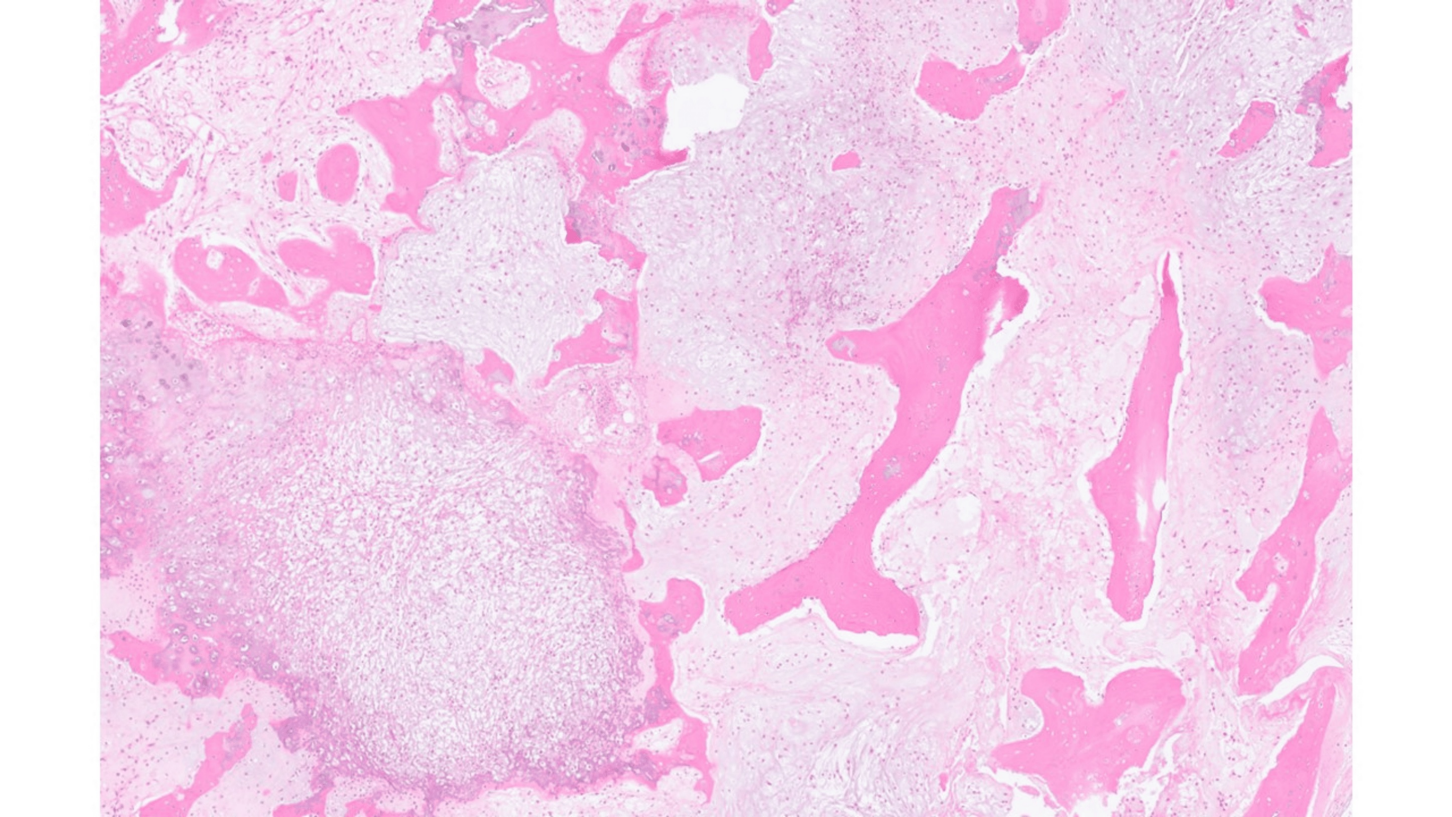
Higher power of Hematoxylin and Eosin (HE) section shows moderately cellular cartilaginous tumour infiltrating pre-existing host bone lamellae. Magnification, digital image: 3.4/43x

**Figure 5:**
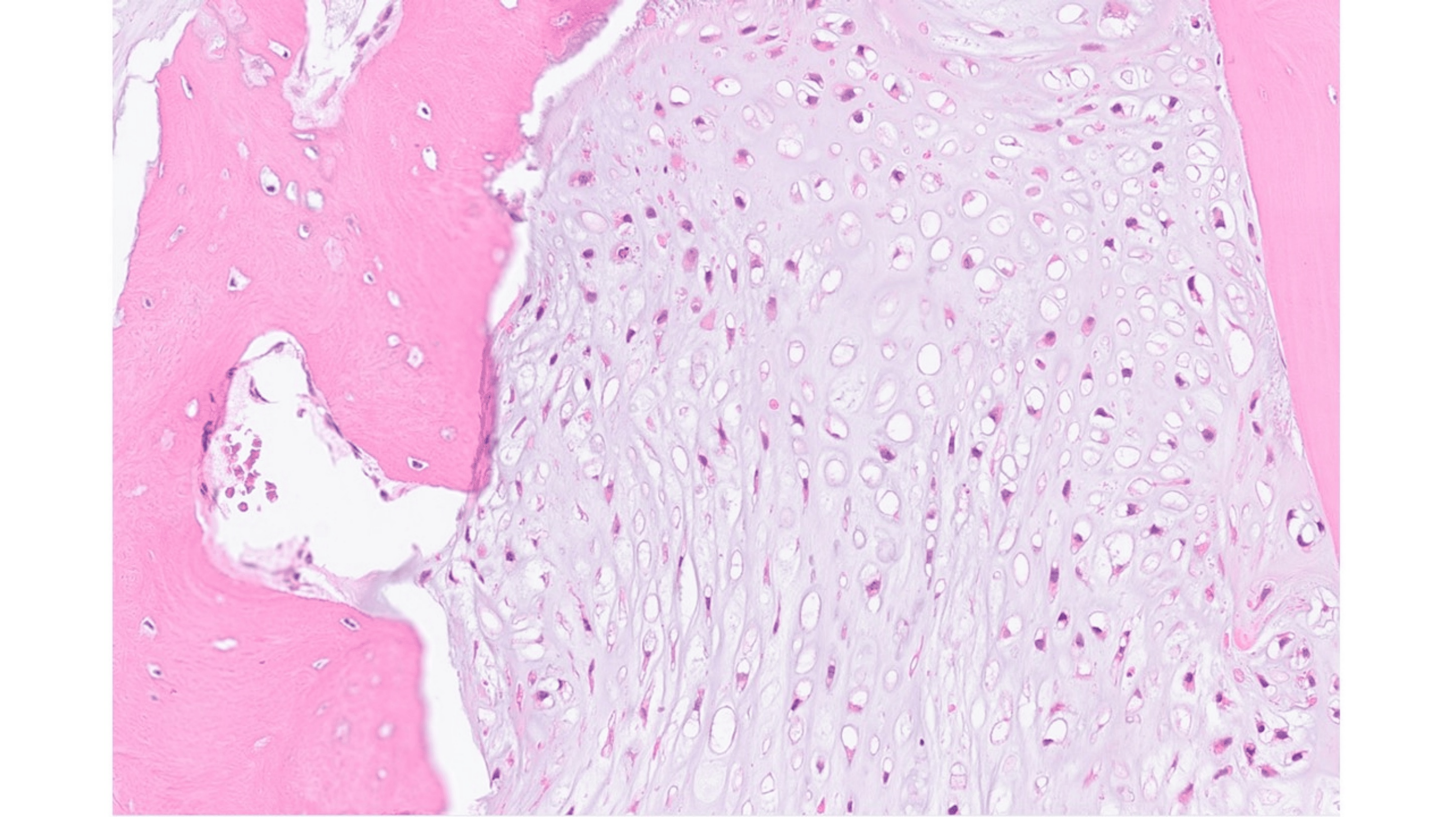
High power Hematoxyling and Eosin (HE) section shows round to spindle shaped chondrocytes with nuclear pleomorphism within background myxoid matrix. Magnification, digital image: 17/43x

The CT-guided core biopsy showed chondrosarcoma of the right calcaneum, grade 1 to 2. There is no evidence of dedifferentiation in the biopsy (Figures 3, 4, 5).

Treatment options

The patient’s case was discussed in the sarcoma multidisciplinary tumour board. Treatment with BKA was recommended. A repeat CT thorax was also arranged. The patient was counselled that if the repeat CT thorax showed stable indeterminate pulmonary nodules or was clear of pulmonary nodules, the BKA would be aimed for curative intent. However, if CT thorax shows progression of lung disease or suspected pulmonary malignancy, then BKA may not be in her best interests, and her treatment will be deferred to symptomatic pain relief medications. The patient agreed to proceed with BKA, which was undertaken on 20th June 2024. The patient is currently on high-grade sarcoma surveillance. The last follow-up was on 15th May 2025. There has been no evidence of local recurrence and no metastases. 

## Discussion

Plantar heel pain is a common complaint affecting both athletes and non-athletes, especially in middle-aged overweight females, accounting for up to 15% of all foot complaints, where the most common cause is known to be plantar fasciitis [19]. Despite many well-established treatment options available for plantar fasciitis, ranging from stretching, taping, to interventions such as shock wave therapy or injections [20], there remains a high risk of recurrence in patients with plantar fasciitis of up to 50% after 5 years and more than 45% after 10 years [21]. This presents a challenge in the diagnosis of recalcitrant heel pain.

The treatment ladder for plantar heel pain has been well established by clinical practice guidelines, which highlights how patients with chronic plantar heel pain should undergo conservative treatment for at least 6 months prior to consideration for more invasive options such as extracorporeal shock wave therapy or fasciotomies [22]. However, there is limited discussion on when to consider further imaging in recalcitrant heel pain when treating patients for plantar fasciitis. This is further compounded by how radiographic imaging is of limited utility in the diagnosis of plantar fasciitis, where plain radiographs are often thought to be unnecessary in the initial evaluation of heel pain, as the majority are either normal or show heel spurs, which do not carry functional significance [23].

There are also ongoing controversies on the diagnostic values of ultrasonography and MRI in plantar fasciitis, but no clear consensus on when to consider further imaging [24]. Whilst MRI is sensitive in the diagnosis of plantar fasciitis, as it identifies other common conditions causing heel pain, including tumours amongst others, such as fascial ruptures, subcalcaneal bursitis, fractures, nerve entrapment syndromes, MRI findings of patients with plantar fasciitis only, such as calcaneal oedema or plantar fascia tears, do not alter management. Hence, MRI is usually not indicated in the investigation of heel pain and is only considered at a later stage when patients do not respond to appropriate treatment [25].

In this study, the 79-year-old female only underwent further imaging after failing conservative treatment for 2 years, which prompts further discussion on when imaging should be pursued. Even though osseous malignancies of the feet are rare, with few cases reported in the literature, there needs to be a high index of suspicion to consider them as a potential diagnosis whenever patients present with non-mechanical pain. In a Mayo Clinic series on over 100 cases of chondrosarcoma of the hands and feet, the calcaneum was the most commonly affected site, affecting 11 patients. Similar to the patient in our case report, the majority of the patients in the clinic series presented with joint pain and swelling, whilst 37% of them presented with swelling without pain, and 24% presented with pain without swelling [26]. 

The majority of chondrosarcomas (90%) arise de novo, such as in this patient. This is differentiated from secondary chondrosarcomas, where peripheral chondrosarcomas arise from osteochondromas as precursor lesions, and central chondrosarcomas arise from enchondromas. Patients with secondary chondrosarcomas are generally younger, with a mean age of 34 years, and patients tend to present with known precursor lesions first, such as a clinically palpable mass. These benign lesions rarely undergo malignant transformation, and even if they do, they usually manifest as low-grade tumours. This is distinct from primary chondrosarcomas, which, depending on their histological subtype, can present as high-grade chondrosarcomas early. The gold standard of distinguishing high-grade chondrosarcomas from low-grade chondrosarcomas, such as atypical cartilaginous tumours (ACT), is an MRI, where the extent of marrow replacement and soft tissue extension is evaluated. This highlights the importance of early imaging: it is important to recognize chondrosarcomas early, as high-grade chondrosarcomas require wide resection with clear surgical margins, whilst low-grade chondrosarcomas such as ACTs may be treated with intralesional curettage and regular surveillance [27].

O​​ther malignant mimickers of plantar fasciitis have also been reported in literature, although rare: Dai et al reported the case of a 58 year-old female in China with unilateral plantar heel pain from bony metastases of her calcaneum as first presentation of lung primary adenocarcinoma, being misdiagnosed as plantar fasciitis for six months [20]; Gao et al also reported the case of a 24-year-old man in China with unilateral plantar pain on a background of plantar injury 4 years ago, who was eventually diagnosed with synovial cell sarcoma but initially treated as plantar fasciitis and subsequently soft tissue infection [28]. We recommend that plain radiographs be performed for persistent heel pain >6 weeks, and MRI should be considered for recalcitrant cases after 3-6 months. 

Even though chondrosarcomas are rare, with around 190 new cases diagnosed each year in the UK, according to the Bone Cancer Research Trust, chondrosarcomas are the most common form of primary bone cancer, comprising 25% of all malignant bone tumours [29]. Early discovery is imperative - when discovered at low grade, the five-year survival rate is significantly higher at 85-100%, compared to when chondrosarcomas are diagnosed at high grade, where the five-year survival rate drops to 27-60% [30]. Surgery is also the only modality of treatment; long-term survival has been shown to be observed only in patients who underwent surgical resection with adequate margins [31]. Ablative surgery may be needed in high-grade foot chondrosarcomas - there is ongoing international debate on what is considered a wide margin needed for high-grade sarcomas as depicted in the Birmingham Orthopaedic Oncology Meeting (BOOM) consensus [32].

Despite the ongoing controversies, it has been shown that achieving at least a 2 mm margin for pelvic high-grade chondrosarcomas is crucial for achieving optimal oncological outcomes [33]. In a large tumour database from the Royal Orthopaedic Hospital for foot malignancies, including that of the calcaneum, surgical amputation was performed for 73% of the patients with primary malignant bone tumour, where 82% of them experienced no local recurrence or metastases. For patients with foot malignancies who had prior initial accidental excisions before being referred to our tertiary centre, the majority of them underwent amputation and achieved a five-year survival rate of 75% with no local recurrence or metastases [18]. 

There has been limited research on the extent to which diagnostic delays impact the survival of patients with chondrosarcoma - in the aforementioned study by Goedhart et al, there was no significant difference in OS between patients who experienced delay from presentation to histological diagnosis of <4 months versus that of ≥4 months [8]. However, in another large-scale Dutch study, the histological grade of chondrosarcoma at diagnosis has been shown to be significantly associated with worse OS, where the 10-year OS for grade I chondrosarcoma was 88%, grade II chondrosarcoma was 62%, and grade III chondrosarcoma was 26% [34]. Even though it is debatable whether diagnostic delays have a long-term impact on OS, timely diagnosis provides better chances for patients to undergo limb-salvage procedures in place of amputations, especially with the advent of modern limb-salvage techniques [35]. According to the BOOM consensus, there seems to be no differences in survival between limb salvage surgery and amputation, as long as wide surgical margins of resection are obtained [31]. 

Unfortunately, in this case report, the diagnostic delays possibly resulted in BKA as the only option for this patient, given the extensive calcaneal destruction present at diagnosis.

## Conclusions

Chondrosarcoma is rare, with associated poor survival when high grade. Surgery is the modality of treatment to obtain a widely clear margin to prevent metastases and local recurrence. Osseous malignancies of the foot, including chondrosarcoma of the calcaneum as depicted in this case, remain rare conditions. Whilst it is challenging to clinically differentiate recalcitrant plantar fasciitis and malignant conditions of the foot, this case report calls for attention to consideration for early imaging in the management of non-mechanical foot pain. We recommend that plain radiographs should be performed for persistent heel pain >6 weeks, and MRI should be considered for recalcitrant cases within 3-6 months.
